# The efficacy of manual therapy (Chuna) for chronic obstructive pulmonary disease: Protocol for a systematic review

**DOI:** 10.1097/MD.0000000000018832

**Published:** 2020-02-28

**Authors:** Ji-Ae Roh, Kwan-Il Kim, Jihye Park, Beom-Joon Lee, Hee-Jae Jung

**Affiliations:** a Department of Clinical Korean Medicine; b Division of Allergy, Immune and Respiratory System, Department of Internal Medicine, College of Korean Medicine; c Department of Biomedical Science and Technology, Graduate School, Kyung Hee University, Dongdaemun-gu, Seoul, Republic of Korea.

**Keywords:** chronic obstructive pulmonary disease, Chuna, manual therapy, protocol, systematic review

## Abstract

**Background:**

Chronic obstructive pulmonary disease (COPD) is a challenging disease whose prevalence has increased over the last decades. Non-pharmacological therapies such as pulmonary rehabilitation occupy a core position in patient management. Among these therapies, manual therapy reduces symptoms and increases exercise capacity targeting musculoskeletal problems. Chuna, a traditional Korean manual therapy, has improved pulmonary disorders in clinical practice but unfortunately has not had its effects reported. This systematic review aims to evaluate the effectiveness and safety of manual therapy, focused on the use of Chuna in the management of COPD patients.

**Methods:**

The following databases will be used in this study: MEDLINE via PubMed, EMBASE, Cochrane Central Register of Controlled Trials (CENTRAL), China National Knowledge Database (CNKI), KoreaMed, Korean Medical Database (KMbase), and Oriental Medicine Advanced Searching Integrated System (OASIS). The primary outcome comprises lung function and exercise capacity. Secondary outcomes included symptoms, quality of life, and adverse events. We will include randomized controlled trials (RCTs) and crossover studies. Two independent reviewers will screen the searched studies, determine if they are suitable for inclusion, and perform data extraction. The risk of bias will be assessed using the Cochrane risk of bias tool. When appropriate, data will be pooled across studies for meta-analysis using a fixed or random effects model. When quantitative synthesis is not appropriate, the evidence will be summarized qualitatively.

**Results:**

This study will provide a comprehensive review of the available evidence to assess the efficacy of Chuna for COPD patients. Results will be published in a peer-reviewed journal and disseminated electronically and in print.

**Conclusions:**

This study will provide high-quality current evidence for evaluation the efficacy of Chuna for patients with COPD. Clinicians, patients and policy makers may find this review useful in making decisions regarding the use of Chuna for patients with COPD.

**PROSPERO registration number:**

CRD42019141150

## Abstract 2

Supplemental Digital Content is available in the text

## 1 Introduction

Chronic obstructive pulmonary disease (COPD) is a common respiratory disease characterized by persistent respiratory symptoms and airflow limitation.^[1]^ It leads to a considerable loss in quality of life and is associated with high morbidity and mortality.^[2,3]^ National Health and Nutrition Examination Survey (NHANES) data from 2007 to 2010 reported that 13.5% of adults aged 20 to 79 years in United States had airflow obstruction.^[4]^ The World Health Organization (WHO) reported that 251 million people worldwide were COPD patients in 2016 and 3.15 million people died by COPD in 2015. The prevalence of COPD has increased over the last decades due to continued exposure to COPD risk factors and global population aging, and this trend is likely to continue in the future.^[5]^

As the burden of COPD increased, the challenge raised a shift in the focus of intervention, from the treatment of shortness of breath to the maintenance of quality of life in order to inhibit the deterioration of lung function. Along with this trend, pulmonary rehabilitation is also important in COPD treatment.^[6]^ Global Initiative for Chronic Obstructive Lung Disease (GOLD) criteria supports that both pharmacologic and nonpharmacologic therapies should be guided by the disease's severity.^[1]^

Pulmonary rehabilitation includes exercise training, education, and behavior changes, designed to improve physical and psychological conditions. It aims to relieve shortness of breath and improve exercise performance to improve quality of life.^[7]^ Physical exercise is the most important feature of pulmonary rehabilitation, and there has been much research on manual therapy targeting the musculoskeletal structure of the thoracic region.^[8]^ COPD patients experience weakening of the skeletal muscle, atrophy, and fiber type deformation, resulting in muscle weakness and reduced oxidative capacity.^[9]^ A great feature of manual therapy is strengthening the patient's respiratory muscles to reduce dyspnea and increase exercise capacity. Several systematic reviews (SRs) have reported the effects of manual therapy on patients with COPD.^[8,10–12]^

Chuna, which is a traditional manual therapy practiced by Korean medicine doctors, has been applied to various diseases in Korea. Chuna manual therapy (CMT) is a technique that uses the hand, other parts of the doctor's body or other supplementary devices such as a table to restore the normal function and structure of pathological somatic tissues by mobilization and manipulation. CMT includes various techniques such as thrust, mobilization, distraction of the spine and joints, and soft tissue release. These techniques were developed by combining aspects of Chinese Tuina, chiropratic, and osteopathic medicine.^[13]^ It has been actively growing in Korea, academically and clinically, since the establishment of the Chuna Society (the Korean Society of Chuna Manual Medicine for Spine and Nerves, KSCMM) in 1991.^[14]^ Recently, Chuna has had its effects nationally recognized and was included in the Korean national health insurance in March 2019.^[15]^

CMT has been used for musculoskeletal disorders in COPD patients and improvements in respiratory function and quality of life have been observed in clinical practice in patients with COPD that receive CMT. However, the effects of CMT on COPD patients have not yet been studied. The previous SRs did not reflect the effects of CMT because they excluded Chinese or Korean database searches, where CMT is frequently used. In this regard, we aim to clarify the scope of the Korean CMT and evaluate the effects and the safety of CMT on COPD patients.

## 2 Methods

### 2.1 Study registration

This protocol for a systematic review has been registered on the international Prospective Register of Systematic Reviews (PROSPERO) with the registration number CRD42019141150, and it will be conducted under the preferred reporting items for systematic reviews and meta-analyses (PRISMA) guidelines.^[25]^

### 2.2 Eligibility criteria

This study has been designed following the PICOS (participant, intervention, comparison, outcome and study design).

#### 2.2.1 Types of studies

All prospective randomized controlled trials (RCTs), quasi-RCTs, and crossover studies will be included in this study. Studies with non-RCTs, studies, case reports, case series, and animal experiments will be excluded.

#### 2.2.2 Types of participants

The global GOLD criteria are a tool for assessing the severity of COPD patients by examining objective pulmonary function and subjective symptoms.^[1]^ Adult patients (over 18 years of age) who were diagnosed with COPD will be included in this study regardless of sex, COPD stage, and history of exacerbations. Patients with COPD having other significant diseases affecting respiratory systems such as lung cancer or other cancers will be excluded from this study. Studies including subjects with COPD as well as other respiratory diseases (e.g., asthma or asthma COPD overlap syndrome, ACOS) will also be excluded.

#### 2.2.3 Types of interventions and controls

Studies reporting any type of manual therapy that is appropriate to CMT such as mobilization, massage, soft tissue therapy, manipulation, chiropractic, ostheopathy, spinal manipulative therapy, and other techniques passively applied by using the practitioners’ hands will be included. Eligible treatments can be used with or without other interventions, but manual therapy techniques should be the main tested intervention. Studies of exercise therapy, acupressure, reflexology, home-based self-treatments, active stretching, and therapies performed by non-specialists will be excluded. Comparators will include placebo, routine pulmonary rehabilitation, non-therapeutic touch, medication, and other positive interventions.

#### 2.2.4 Types of outcome measures

##### 2.2.4.1 Primary outcome

(1) Lung function
  We will include a lung function parameter, such as forced expiratory volume in 1 second (FEV_1_), forced vital capacity (FVC), FEV_1_/FVC, or vital capacity (VC).
(2) Exercise capacity (6-minute walk distance, 6MWD)
  The 6MWD test considers the time spent walking a distance of 30 m, and objectively measures the athletic performance of patients with COPD. Walking tests are useful in assessing activity limitations and risk of death.^[16]^

##### 2.2.4.2 Secondary outcomes

(1) Symptoms
  We will include all COPD symptoms described in the studies. The severity of dyspnea will be assessed using the modified Medical Research Council (mMRC) dyspnea scale developed in England. If mMRC is not used, another assessment tool such as patient-reported measures, self-assessment, and/or questionnaires can be used.
(2) Quality of life:
  Quality of life will be assessed using the COPD assessment test (CAT). CAT is useful for assessing quality of life, including respiratory symptoms other than dyspnea, activity, sleep, and self-confidence in daily life. If CAT is not used, another assessment tool such as the St. George respiratory questionnaire (SGRQ) can be used.
(3) Adverse events or safety measurement.

### 2.3 Search methods for identifying studies

#### 2.3.1 Electronic searches

Electronic databases including MEDLINE via PubMed, EMBASE, Cochrane Central Register of Controlled Trials (CENTRAL), and China National Knowledge Database (CNKI) will be searched from inception without language restrictions. Three Korean medical databases including KoreaMed, Korean Medical Database (KMbase), and Oriental Medicine Advanced Searching Integrated System (OASIS) will also be searched. In addition, other resources such as the references of all included studies will be searched manually.

#### 2.3.2 Other sources

The reference lists of the retrieved articles, relevant systematic reviews, and unpublished conference proceedings (if available) will be searched manually. Ongoing RCT registers such as clinicaltrials.gov and WHO International Clinical Trials Registry Platform (WHO ICTRP) will be reviewed and the information that cannot be verified should be confirmed by contacting the study authors, if possible.

#### 2.3.3 Search strategy

Search terms will be related to disease and intervention. Related Medical Subject Heading (MeSH) terms and synonyms in various combinations will be used as search strategies. The terms to be used in relation to the disease include “COPD,” “emphysema,” “bronchitis.” The terms to be used in relation to the intervention include “chuna (tuina),” “manual therapy,” “manipulation,” “mobilization.” The search strategies are presented in online Supplemental Digital Content (Appendix 1–4).

### 2.4 Data collection and analysis

#### 2.4.1 Study inclusion and selection of studies

**Figure 1:**
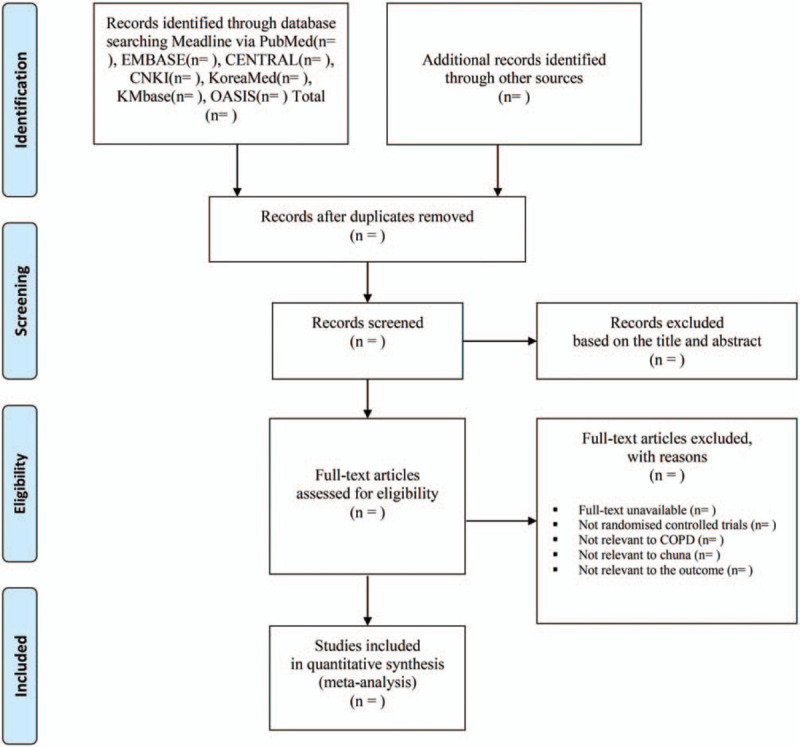
Flowchart of identification and screening for the eligible studies.

Two reviewers (JAR and KIK) will independently screen the titles and abstracts of the searched studies to check for eligibility based on the PICOS. The full text will also be reviewed by the 2 independent reviewers (JAR and KIK) before inclusion. All studies electronically and manually searched will be uploaded to EndNote X9.2 (Clarivate Analytics, Philadelphia, PA) for bibliographic management and review. The detailed study selection procedure is presented in the PRISMA flow chart (Fig. 1). Discrepancies will be resolved by discussion with a third researcher (HJJ) to determine, by agreement, the final selection of studies.

#### 2.4.2 Data extraction and management

Two of the reviewers (JAR and KIK) will independently extract data using a standard data extract form (a pre-designed Microsoft Excel spreadsheet). The form includes general study information (publication year, first author, country, and title), study characteristics (study design, allocation ratio, number of groups, randomization, blinding, and number of withdrawals and dropouts), inclusion/exclusion criteria, COPD diagnosis criteria, number of participants, patients’ age, sex, COPD stage, pattern type based on traditional Korean (TKM) or Chinese medicine (TCM), interventions (type of intervention, number of treatments, treatment period, total number of treatments, practitioner type), comparators, outcomes (primary and secondary), adverse events. Any discrepancies in the data will be reviewed by another researcher (HJJ).

### 2.5 Assessment of risk of bias in the included studies

Two reviewers (JAR and KIK) will independently assess the risk of bias according to the Cochrane Handbook 5.1.0. assessment tool.^[17]^ This tool comprises on the following categories: random sequence generation, allocation concealment, blinding of participants and personnel, blinding of outcome assessment, incomplete outcome data, selective outcome reporting, and other sources of bias (baseline imbalance).

Disagreements will be resolved by consulting with another researcher (BJL). When necessary, study authors will be contacted to verify any missing information.

### 2.6 Strategy for data synthesis and statistical analysis

#### 2.6.1 Dealing with missing data

For included studies in which there are missing data or the analysis process is unclear, the associated risk of bias will be fully considered. The authors will be contacted via email or telephone about information that is not available in the study. If data are still insufficient after contacting the author, it will be analyzed using the available data.

#### 2.6.2 Data synthesis

Data analysis will be performed using Review Manager Software (RevMan, Version 5.3 for Windows; The Nordic Cochrane Centre, The Cochrane Collaboration, Copenhagen, 2014). The mean difference, confidence interval (CI), risk ratio, and the *P*-values will be used to evaluate the effectiveness and safety of Chuna therapy. The weighted mean difference and the 95% CI will be calculated for continuous data collected using the same measurement scale. The standardized mean differences with 95% CI will be calculated for continuous data collected using different measurement scales for the same outcome. The risk ratio with 95% CI and *P*-values will be used for dichotomous outcomes. When appropriate, data will be pooled across studies for meta-analysis using fixed or random effect models. When quantitative synthesis is not appropriate due to heterogeneity, we will offer summary tables of study characteristics and outcome measures and do a narrative synthesis.

#### 2.6.3 Assessment of heterogeneity

An *I*^2^ test with a significance level of *P* < .1 will be used to assess inconsistencies among studies. The *I*^2^ statistic indicates the proportion of variability amongst studies that is not explained by chance alone and an *I*^2^ value ≥50% is considered to be an indicator of substantial heterogeneity.^[18]^ The fixed-effects model will be applied to homogeneous data (*I*^2^ value < 50%), and if *I*^2^ value ≥50% (*P*-value < .10), the random-effects model will be applied.

#### 2.6.4 Assessment of reporting biases

If the analysis includes ≥10 studies in meta-analysis, a funnel plot will be used to detect publication bias.^[19,20]^

#### 2.6.5 Subgroup analysis and investigation of heterogeneity

If there is an adequate number of studies, we will conduct subgroup analyses to interpret the heterogeneity between studies. Subgroup analyses will be performed based on COPD severity according to GOLD criteria (e.g., stages 1 or 2 vs stages 3 or 4), duration of follow up (e.g., immediate, mid-term, or long-term), intervention type (e.g., monotherapy or multiple therapies, mobilization, or manipulation), and control intervention type (e.g., placebo, sham-touch, medication, other rehabilitation, or no treatment). If possible, subgroup analyses will also be conducted for pattern identification and clinical judgment.

#### 2.6.6 Sensitivity analysis

Sensitivity analysis will be performed to investigate the robustness of the selected studies. The “risk of bias” tool will also be used to evaluate the quality of the studies’ methodology. Studies that are rated low grade over 3 items will be excluded. The meta-analysis will be repeated to once again identify whether the methodological quality, sample size, and missing information influence the review's findings. The results of the sensitivity analysis will be reported in a summary table.

#### 2.6.7 Grading the quality of evidence

The Grading of Recommendations Assessment, Development and Evaluation (GRADE) method will be used to assess the level of evidence concerning main outcomes and recommendation strength of related treatments^[21]^ by 2 independent reviewers (JAR and KIK) and any discrepancies will be resolved by another researcher (HJJ).

### 2.7 Ethics and dissemination

Ethical approval is not required. We will review the published studies, and since there is no patients involved in this study. Results will be published in a peer-reviewed journal and disseminated electronically and in print.

## 3 Discussion

This study is the first research protocol to systematically review the efficacy and safety of CMT on COPD patients. Traditional Korean Chuna is based on traditional Chinese Tuina, whereas current CMT is a modernized technique that integrates traditional practice and modern scientific theory. Therefore, it bears similarities with Tuina, chiropractic practice, and osteopathy, and among other manual therapies it includes techniques such as thrust, mobilization, distraction of the spine and joints, visceral manipulation, soft tissue release, and craniosacral therapy.^[13,22]^

There is much evidence for the impaired physical activity and dyspnea observed in COPD patients, which could be induced by musculoskeletal disorders and pain.^[23]^ Exercise intolerance in COPD patients can be explained by skeletal and respiratory muscle dysfunction. A reduced aerobic metabolism in skeletal muscles such as the quadriceps increases lactic acidosis and aggravates ventilatory demand. Hyperinflation, which is very common in COPD, damages the inspiratory muscle function resulting in respiratory muscle weakness.^[24]^ Chuna can address the problems regarding musculoskeletal and respiratory systems by manipulating the structures in the thoracic region.

The effect of manual therapies equivalent to Chuna in COPD has not yet been systematically studied. Patients whose symptoms cannot be managed by routine therapy alone may benefit from CMT. With as much current evidence as possible, all the techniques included in the definition of or corresponding to Chuna will be searched.

Manual therapies have been reported as controversial or beneficial for COPD in systemic reviews. However, this evidence has limitations in that it does not include studies from East Asian countries where traditional manual therapy is frequently used. This study may clarify the effectiveness of CMT in COPD treatment, avoiding the bias in search coverage and enabling access to studies originally published in East Asian languages, which would otherwise be inaccessible due to language barriers.

This study will provide updates on RCTs and broaden the search coverage in relation to previous studies. This systematic review will involve the extensive and unbiased review of 7 databases including Chinese and Korean databases. We also followed the PRISMA-P checklist.^[25]^ The results should be cautiously considered because the strength of the evidence relies on both quality and quantity of original research exploring the issue in a robust way. In addition, it is also necessary to be cautious regarding the heterogeneity of the types of interventions.

A systematic review with good quality can help provide further evidence regarding the effectiveness and safety of CMT in improving lung function, exercise capacity, symptoms, and quality of life of patients with COPD. This review will help clinicians, patients, and policy makers to make better decisions regarding the appropriate role of Chuna therapy as a part of patient management routines such as pulmonary rehabilitation, physiotherapy, or complementary and alternative medicine.

## 4 Conclusion

The conclusion of this study will provide evidence to confirm whether CMT can be safely and effectively applied to patients with COPD.

## Author contributions

**Conceptualization:** Hee-Jae Jung, Beom-Joon Lee

**Data curation:** Ji-Ae Roh, Kwan-Il Kim

**Formal analysis:** Ji-Ae Roh, Jihye Park

**Investigation:** Ji-Ae Roh, Jihye Park

**Methodology:** Kwan-Il Kim, Beom-Joon Lee

**Writing – original draft:** Ji-Ae Roh, Kwan-Il Kim

**Writing – review & editing:** Kwan-Il Kim, Hee-Jae Jung

Hee-Jae Jung orcid: 0000-0001-7384-6881

Beom-Joon Lee orcid: 0000-0003-4205-1175

**Kwan-Il Kim orcid:** 0000-0003-4205-1175

**Ji-Ae Roh orcid:** 0000-0001-5125-0999

## Supplementary Material

Supplemental Digital Content
