# Supplementary material for: The efficacy of manual therapy (Chuna) for chronic obstructive pulmonary disease: Protocol for a systematic review
Source: Medicine (Baltimore). 2020 Feb 28;99(9):e18832. doi: 10.1097/MD.0000000000018832 (PMC7478514; doi:10.1097/MD.0000000000018832)
Supplement: Supplemental Digital Content [file medi-99-e18832-s001.docx]

**Appendix 1. Search strategies**

**Table 1. The Cochrane Library**

| #1 | MeSH descriptor: [Pulmonary Disease, Chronic Obstructive] explode all trees |
| --- | --- |
| #2 | MeSH descriptor: [Bronchitis, Chronic] explode all trees |
| #3 | MeSH descriptor: [Pulmonary Emphysema] explode all trees |
| #4 | (obstruct*) near/3 (pulmonary or lung* or airway* or airflow* or bronch* or respirat*) |
| #5 | (COPD or COAD or COBD or AECB or AECOPD):ti,ab,kw |
| #6 | #1 or #2 or #3 or #4 or #5 |
| #7 | MeSH descriptor: [Chiropractic] explode all trees |
| #8 | MeSH descriptor: [Osteopathic Medicine] explode all trees |
| #9 | MeSH descriptor: [Orthopedics] explode all trees |
| #10 | MeSH descriptor: [Manipulation, Spinal] explode all trees |
| #11 | MeSH descriptor: [Manipulation, Chiropractic] explode all trees |
| #12 | MeSH descriptor: [Manipulation, Osteopathic] explode all trees |
| #13 | MeSH descriptor: [Manipulation, Orthopedic] explode all trees |
| #14 | MeSH descriptor: [Musculoskeletal Manipulations] explode all trees |
| #15 | MeSH descriptor: [Therapy, Soft Tissue] explode all trees |
| #16 | MeSH descriptor: [Massage] explode all trees |
| #17 | (physical therap*):ti,ab,kw |
| #18 | (physiotherap*):ti,ab,kw |
| #19 | (manual near/2 therap*):ti,ab,kw |
| #20 | Musculoskeletal Manipulat* |
| #21 | (mobili?ation):ti,ab,kw |
| #22 | (manipulat*):ti,ab,kw |
| #23 | Spin* near/4 manipulat* |
| #24 | Chiroprac* |
| #25 | Osteopath* |
| #26 | Orthopedic Manipulat* |
| #27 | Muscle next energy next technique |
| #28 | (Muscle near/4 stretching):ti,ab,kw |
| #29 | Post isometric relaxation |
| #30 | (Isometric next contraction):ti,ab,kw |
| #31 | (Isometric stretching):ti,ab,kw |
| #32 | Proprioceptive neuromuscular facilitation |
| #33 | (Myofascial release):ti,ab,kw |
| #34 | (massag*):ti,ab,kw |
| #35 | "Tui Na" or "Tuina" or "Chuna" |
| #36 | Tuina |
| #37 | Chuna |
| #38 | #7 or #8 or #9 or #10 or #11 or #12 or #13 or #14 or #15 or #16 or #17 or #18 or #19 or #20 or #21 or #22 or #23 or #24 or #25 or #26 or #27 or #28 or #29 or #30 or #31 or #32 or #33 or #34 or #35 or #36 |
| #39 | #6 and #38 |
| #40 | MeSH descriptor: [Animals] explode all trees |
| #41 | #39 not #40 |
|  |  |

**Appendix 2. Search strategies**

**Table 2. Medline (PubMed)**

| #1 | Pulmonary disease, chronic obstructive[mh] |
| --- | --- |
| #2 | Bronchitis, chronic[mh] |
| #3 | pulmonary emphysema[mh] |
| #4 | Emphysema*[tiab] |
| #5 | Obstruct*[tiab] and Pulmonary[tiab] |
| #6 | chronic bronchiti*[tiab] |
| #7 | Obstruct*[tiab] and Lung[tiab] |
| #8 | Obstruct*[tiab] and Airway[tiab] |
| #9 | Obstruct*[tiab] and Airflow[tiab] |
| #10 | Obstruct*[tiab] and Bronchial[tiab] |
| #11 | Obstruct*[tiab] and Respiratory[tiab] |
| #12 | COPD[tiab] |
| #13 | COAD[tiab] |
| #14 | COBD[tiab] |
| #15 | AECB[tiab] |
| #16 | #1 or #2 or #3 or #4 or #5 or #6 or #7 or #8 or #9 or #10 or #11 or #12 or #13 or #14 or #15 |
| #17 | Chiropractic[mh] |
| #18 | Osteopathic medicine[mh] |
| #19 | Orthopedics[mh] |
| #20 | Manipulation, spinal[mh] |
| #21 | Manipulation, chiropractic[mh] |
| #22 | Manipulation, osteopathic[mh] |
| #23 | Manipulation, orthopedic[mh] |
| #24 | Musculoskeletal manipulations[mh] |
| #25 | Therapy, soft tissue[mh] |
| #26 | Massage[mh] |
| #27 | Manual therap*[tiab] |
| #28 | Physical therap*[tiab] |
| #29 | Physiotherap*[tiab] |
| #30 | Musculoskeletal manipulat*[tiab] |
| #31 | Manipulat*[tiab] |
| #32 | Mobilization*[tiab] |
| #33 | Mobilisation*[tiab] |
| #34 | Spine manipulat*[tiab] |
| #35 | Spinal manipulat*[tiab] |
| #36 | Chiropractic[tiab] |
| #37 | Osteopath*[tiab] |
| #38 | Orthopedic manipulat*[tiab] |
| #39 | Muscle energy technique*[All Fields] |
| #40 | Muscle stretching[tiab] |
| #41 | Post isometric relaxation[tiab] |
| #42 | Isometric contraction[tiab] |
| #43 | Isometric stretching[All Fields] |
| #44 | Proprioceptive neuromuscular facilitation[All Fields] |
| #45 | Myofascial release[tiab] |
| #46 | Massag*[tiab] |
| #47 | "Tui Na"[All Fields] |
| #48 | Tuina[All Fields] |
| #49 | Chuna[All Fields] |
| #50 | #17 or #18 or #19 or #20 or #21 or #22 or #23 or #24 or #25 or #26 or #27 or #28 or #29 or #30 or #31 or #32 or #33 or #34 or #35 or #36 or #38 or #39 or #40 or #41 or #42 or #43 or #44 or #45 or #46 or #47 or #48 or #49 |
| #51 | #16 and #50 |
| #52 | Randomized controlled trial[pt] or Controlled clinical trial[pt] or Randomized[tiab] or Placebo[tiab] or Drug therapy[sh] or Randomly[tiab] or Trial[tiab] or Groups[tiab] not (Animals [mh] not Humans [mh]) |
| #53 | #51 and #52 |
| #54 | #53 and Humans[mh] |
| #55 | #54 and Clinical Trial[ptyp] |
|  |  |

**Appendix 3. Search strategies**

**Table 3. EMBASE**

| #1 | 'chronic obstructive lung disease'/exp |
| --- | --- |
| #2 | 'chronic bronchitis'/exp |
| #3 | 'lung emphysema'/exp |
| #4 | emphysema$:ti,ab,kw |
| #5 | (chronic$ NEXT/3 bronchiti*):ti,ab,kw |
| #6 | (obstruct* NEXT/3 (pulmonary OR lung$ OR airway$ OR airflow$ OR bronch* OR respirat*)):ti,ab,kw |
| #7 | copd:ti,ab,kw |
| #8 | coad:ti,ab,kw |
| #9 | cobd:ti,ab,kw |
| #10 | aecb:ti,ab,kw |
| #11 | #1 or #2 or #3 or #4 or #5 or #6 or #7 or #8 or #9 or #10 |
| #12 | 'chiropractic manipulation'/exp |
| #13 | 'osteopathic manipulation'/exp |
| #14 | 'orthopedic manipulation'/exp |
| #15 | 'spine manipulation'/exp |
| #16 | 'musculoskeletal manipulation'/exp |
| #17 | 'chiropractic'/exp |
| #18 | 'osteopathic medicine'/exp |
| #19 | 'soft tissue therapy'/exp |
| #20 | 'massage'/exp |
| #21 | 'joint mobilization'/exp |
| #22 | 'physiotherapy'/exp |
| #23 | 'muscle isometric contraction'/exp |
| #24 | 'isometrics'/exp |
| #25 | 'physical therap*':ti,ab,kw |
| #26 | physiotherap*:ti,ab,kw |
| #27 | (manual NEAR/2 therap*):ti,ab,kw |
| #28 | 'musculoskeletal manipulat*':ti,ab,kw |
| #29 | manipulat*:ti,ab,kw |
| #30 | mobili?ation:ti,ab,kw |
| #31 | (spin* NEAR/4 manipulat*):ti,ab,kw |
| #32 | chiroprac*:ti,ab,kw |
| #33 | osteopath*:ti,ab,kw |
| #34 | 'orthopedic manipulat*':ti,ab,kw |
| #35 | 'muscle energy technique$':ti,ab,kw |
| #36 | 'post$isometric relaxation':ti,ab,kw |
| #37 | 'isometric stretching':ti,ab,kw |
| #38 | 'isometric contract*':ti,ab,kw |
| #39 | 'proprioceptive neuromuscular facilitation':ti,ab,kw |
| #40 | 'myofascial release':ti,ab,kw |
| #41 | massag*:ti,ab,kw |
| #42 | 'tui na':ti,ab,kw |
| #43 | tuina:ti,ab,kw |
| #44 | chuna:ti,ab,kw |
| #45 | #12 or #13 or #14 or #15 or #16 or #17 or #18 or #19 or #20 or #21 or #22 or #23 or #24 or #25 or #26 or #27 or #28 or #29 or #30 or #31 or #32 or #33 or #34 or #35 or #36 or #37 or #38 or #39 or #40 or #41 or #42 or #43 or #44 |
| #46 | #11 and #45 |
| #47 | #46 and ([controlled clinical trial]/lim OR [randomized controlled trial]/lim) and [humans]/lim |
|  |  |

**Appendix 4. Search strategies**

**Table 4. CNKI**

| 1 | COPD |
| --- | --- |
| 2 | 慢性阻塞性肺疾病 |
| 3 | 慢性阻塞性肺病 |
| 4 | 慢性阻塞性肺 |
| 5 | 肺气肿 |
| 6 | 慢性支气管炎 |
| 7 | 慢性气管炎 |
| 8 | 慢阻肺疾病 |
| 9 | 慢阻肺病 |
| 10 | 慢阻肺 |
| 11 | #1 or #2 or #3 or #4 or #5 or #6 or #7 or #8 or #9 or #10 |
| 12 | 手法治疗 |
| 13 | 推拿 |
| 14 | 推拿治疗 |
| 15 | 推拿疗法 |
| 16 | 推拿手法 |
| 17 | 推拿手法治疗 |
| 18 | 推拿正骨 |
| 19 | 理疗 |
| 20 | 理疗治疗 |
| 21 | 物理疗法 |
| 22 | 脊柱推拿手法治疗 |
| 23 | 捏脊 |
| 24 | 捏脊疗法 |
| 25 | 正骨手法 |
| 26 | 正骨手法治疗 |
| 27 | 正骨疗法 |
| 28 | 正骨推拿 |
| 29 | 正骨推拿治疗 |
| 30 | 正骨推拿手法 |
| 31 | 正骨理筋手法 |
| 32 | 整骨疗法 |
| 33 | 整骨手法 |
| 34 | 整骨技术 |
| 35 | 整骨医学 |
| 36 | 整骨理筋手法 |
| 37 | 理筋正骨法 |
| 38 | 理筋正骨手法 |
| 39 | 理筋手法 |
| 40 | 肌肉能量技术 |
| 41 | 等距 |
| 42 | 关节松动术 |
| 43 | 关节松动 |
| 44 | 筋膜松动术 |
| 45 | 经筋推拿 |
| 46 | #12 or #13 or #14 or #15 or #16 or #17 or #18 or #19 or #20 or #21 or #22 or #23 or #24 or #25 or #26 or #27 or #28 or #29 or #30 or #31 or #32 or #33 or #34 or #35 or #36 or #37 or #38 or #39 or #40 or #41 or #42 or #43 or #44 or #45 |
| 47 | #11 and #46 |
